# Old Fashioned Drugs

**Published:** 1916-05

**Authors:** G. Henri Bogart

**Affiliations:** Shelbyville, Illinois


### Old Fashioned Drugs.

BY DR. G. HENRI BOGART, SHELBYVILLE, ILLINOIS.

A little while ago, only it seems long, long since, we learned from text-book and professor of a class of remedies, called alteratives, something that in some mysterious manner altered the course of vital functions. The great five alteratives were mercury, arsenic, rodine, sulphur, and quinine, the first a specific against syphilis and the last against malaria.

The word specific was a red rag; it was branded as unscientific, and there are many who do not like to hear about specifics, even now. They—the objectors—will wax mightily indignant at the use of the word, but so long as there is a known specific cause, it follows, logically, that there is a specific remedy.

There is an immense lot of bosh about "scientific medication."

The origin of all of it was in trial and tabulated results of experiments somewhere down in the dim morning of the race. It is wonderful beyond understanding how those pioneers of healing discovered and applied these five sheet anchors, how they learned of them, and all of them.

We know now why these drugs are alterative. The diseases which they control are caused by germs, and their action is because they are more toxic to the invading organism than they to the cells of the host.

Salvarsan is known as 606 because it is the result of Erlich's six hundred and sixth experiment. Erlich was seeking a combination of arsenic by which the human organism could withstand a dosage of the remedy in sufficient quantity to destroy, or at least weaken, the spirida palida without bad fatal action on the life cells of the body.

It is difficult to grasp the patient labor necessary to make the 605 arsenical combinations and test them one by one on their relative toxic effects on the human organism and the syphilis germ, but that patient, plodding tenacity finally won a new remedy, which, while it may not be a specific cure, is at least a valuable aid to cure.

But prior to 1880 when Koch determined to a certainty the tubercular bacillus, our uses of the alteratives or germicides was a blind groping in the dark. Knowing the limitations of the art of healing, the medical profession as a class is anxious, and properly anxious, to try every new thing that shall increase the powers of healing.

But time and practice had showed the value of the hoary alteratives and science has come to reinforce their claims by telling us how they act, just as the chemists organized the lore of the alchemists and astronomers built from astrology.

One can use rational deductions with these patriarchs of the materia medica. For instance, it was long ago recognized that in some manner a rheumatism, beginning with the left knee, was associated with gonorrhea, then the germ of Neisser was found and studied. Constitutional gonorrhea is now a recognized condition, though few, too few general practitioners pay much attention to its ravages.

Others, regarding the discoveries of Noguchi, of specific inflammations of serous cavities with their abundant fatalities and their infections of innocent and virtuous companions voice the pessimistic cry that gonorrhea is as persistent as syphilis. I hold that the province of the physician is both cure and prevention. An enemy is more easily routed before he has entrenched himself.

Now I expect to hear an objection that my treatment is routine, but I know that days follow nights in routine, and, besides, the doctor's duty is healing. So when I am called to treat a case of gonorrhea I expect to attempt a cure, and to prevent a lodgment of constitutional complication.

I administer calcium sulphide and arsenic sulphide, to saturation to forestall any stray diplococci, and before using any local urethral applications I fill the urethra with hydrogen peroxide. This dissolves all pus and carries away all free germs and leaves the mucous surfaces clean so that the remedy can act directly.

I am mentioning this routine treatment, not as something startling but as an example of the application of plain, every-day reason for a routine treatment with the time-tried and tested alteratives.

